# Comparative analysis of titanium coating on cobalt-chrome alloy in vitro and in vivo direct metal fabrication vs. plasma spraying

**DOI:** 10.1186/s13018-020-02108-4

**Published:** 2020-11-26

**Authors:** Dongwhan Suh, Woo Lam Jo, Seung Chan Kim, Yong Sik Kim, Soon Yong Kwon, Young Wook Lim

**Affiliations:** 1 Department of Orthopaedic Surgery, College of Medicine, The Catholic University of Korea, Seoul, Republic of Korea; 2 Department of Orthopaedic Surgery, Daejeon St. Mary’s Hospital, College of Medicine, The Catholic University of Korea, Seoul, Republic of Korea; 3 Department of Orthopaedic Surgery, Seoul St. Mary’s Hospital, College of Medicine, The Catholic University of Korea, Seoul, Republic of Korea; 4 Department of Orthopaedic Surgery, Eunpyeong St. Mary’s Hospital, College of Medicine, The Catholic University of Korea, Seoul, Republic of Korea

**Keywords:** Titanium surface coating, Direct metal fabrication, Osteointegration, 3D printing

## Abstract

**Background:**

Titanium surface coating on cobalt-chromium (CoCr) alloy has characteristics desirable for an orthopedic implant as follows: strength, osteointegrative capability, and biocompatibility. Creating such a coated surface takes a challenging process and two dissimilar metals are not easily welded. In our study, we utilized additive manufacturing with a 3D printing called direct metal fabrication (DMF) and compared it to the plasma spraying method (TPS), to coat titanium onto CoCr alloy. We hypothesized that this would yield a coated surface quality as acceptable or better than the already established method of plasma spraying. For this, we compared characteristics of titanium-coated surfaces created by direct metal fabrication method (DMF) and titanium plasma spraying (TPS), both in vitro and in vivo, for (1) cell morphology, (2) confocal microscopy images of immunofluorescent assay of RUNX2 and fibronectin, (3) quantification of cell proliferation rate, (4) push-out biomechanical test, and (5) bone histomorphometry.

**Method:**

For in vitro study, human osteoblast cells were seeded onto the coated surfaces. Cellular morphology was observed with a scanning electron microscope. Cellular proliferation was validated with ELISA, immunofluorescent assay. For in vivo study, coated rods were inserted into the distal femur of the rabbit and then harvested. The rods were biomechanically tested with a push-out test and observed for histomorphometry to evaluate the microscopic bone to implant ratio.

**Result:**

For cell morphology observation, lamellipodia and filopodia, a cytoplasmic projection extending into porous structure, formed on both surfaces created by DMF and TPS. The proliferation of the osteoblasts, the DMF group showed a better result at different optic density levels (*p* = 0.035, 0.005, 0.001). Expression and distribution of fibronectin and Runx-2 genes showed similar degrees of expressions. The biomechanical push-out test yielded a similar result (*p* = 0.714). Histomorphometry analysis also showed a similar result (*p* = 0.657).

**Conclusion:**

In conclusion, DMF is a method which can reliably create a proper titanium surface on CoCr alloy. The resulting product of the surface shows a similar quality to that of the plasma spraying method, both in vivo and in vitro, in terms of biological and mechanical property.

## Introduction

Strength, osteointegrative capability, and biocompatibility are the qualities desirable for an orthopedic implant [1]. Until now, endeavor to meet the optimal surface condition for implants to incorporate such characteristics is ongoing [2]. While it has been known that titanium surface coating on cobalt-chromium (CoCr) alloy would yield such quality [3–5], this process had remained challenging [6]. To create or combine metal on the metal surface, casting or forging plus additive manufacturing processes are required. The additive manufacturing process is known to be dependent on types of metals, and titanium itself poses a challenge to the process due to its high melting point and chemical composition [7–9]. The configuration of the end-product, whether there be a groove or an angle is a significant variable to the process [8]. Thus, it had been considered as a challenge to create cobalt-chromium and titanium alloy with conventional techniques [4].

Plasma spraying is an established, commercially available method used in the additive manufacturing (AM) process. It has been known for its versatility in application and availability [10, 11]. However, this method could cause (1) structural deformation, (2) delamination of the coated surface, (3) non-optimal porosity, (4) decrement in fatigue strength, and (5) relatively high-cost and complexity of the process [8]. Direct metal fabrication (DMF) technique, on the other hand, is thought to be able to overcome the aforementioned shortcomings of the plasma spraying method by minimally affecting the surrounding materials; without creating a wide surface of thermal alteration and extensive weld line [6, 12–14]. Also, DMF does not require vacuum conditions or other types of conditioning prior to application. Thus, the range of applicability could be wider with this method [12].

DMF is a novel, additive manufacturing (AM) method that utilizes 3D printing technology [15]. With a fully automated process, the surface quality and characteristics can be controlled to customize the desired configuration, pore size, and surface roughness [16]. The method allows a stable coating-substrate interface between different physical and chemical properties [17]. We hypothesized that DMF could produce titanium-coated surface as good or better than the already established plasma spraying method. To test our hypothesis, we compared titanium coating with DMF and titanium plasma spraying (TPS) on CoCr alloy surface both in vitro and in vivo for (1) cell morphology, (2) confocal microscopy images of immunofluorescent assay of RUNX2 and fibronectin, (3) quantification of cell proliferation rate through ELISA, (4) interfacial shear strength (push-out biomechanical test), and (5) bone histomorphometry.

## Method

We compared DMF and titanium plasma spray (TPS) coatings of CoCr alloy surface both in vitro and in vivo, to find if there is a difference in terms of cell morphology, biocompatibility, cell proliferation rate, shear strength, and histomorphometry. For in vitro study, with AM technology-based DMF method, Pure Ti (CPTi powder grade 2, ASTM F1580) powders between the size of 45–150 um were melted and laminated using a selective laser on CoCr alloy surface. A computer-assisted design (CAD) program was used prior to executing the actual coating process to design porous structure to simulate the porous properties of cancellous bone (NX-based coating CAM for Insstek, Siemens). The laser-irradiated surface of CoCr alloy formed a melted pool, by following the path of a pre-programmed grid-shaped tool with 80 W laser power, 1.5 m/min scan speed, and 2.2 g/min power delivery rate. Next, metal powders were sprayed and laminated onto the melted surface, which is different from selective laser melting (SLM) and electron beam melting (EBM) [15]. To give the porous surface an irregularity of thickness and shape, the coating layer was twice coated; once with a thickness of 300 um and then with a thickness of 500 um. In the plasma spraying method which we utilized to compare to DMF, Ti powder for coating was injected into plasma gas stream which is heated up to 20,000 °C. With high kinetic force, the powder was shot onto the substrate and then melted, forming a porous structure. Scanning electron microscopy was used to assess the structure and morphology of the produced surfaces [11].

Osteoblasts derived from human mesenchymal cells were prepared [18]. 5 × 10^4^ osteoblasts were seeded onto DMF- and TPS CoCr-coating specimens. After 6 h of seeding of cells in each implant, the media was removed and then the cells were washed with PBS 3 times. After adding 2% glutaraldehyde-PBS solution, these cells were stabilized for 2 h. The cells were then washed with dextrose water solution 3 times. At 30-min intervals, the cells were dehydrated with 50–100% ethanol solutions. The ethanol was removed, and the cells were left at room temperature to allow for complete ethanol evaporation. Two surfaces were then characterized by scanning electron microscope (JEOL JSM-6700F; JEOL, Ltd., Tokyo, Japan) after the test specimens had been coated with platinum.

The seeded cells on the coated surfaces were incubated for 24, 48, 72, and 96 h. The medium was replaced with a fresh medium before measuring cell proliferation using the Cell Titer 96 Nonradioactive Cell Proliferation Assay (Promega Corp, Madison, WI), according to the manufacturer’s instructions. Cell proliferation assay is a colorimetric method for determining the number of viable cells. In this study, the number of viable cells was measured at 490 nm using an enzyme-linked immunosorbent assay (ELISA) reader (Bio-Tek Instruments, Inc., Winooski, VT) [19].

The differentiation of osteoblast cells was evaluated by immunofluorescence staining for the Runx-2 and fibronectin genes [5, 20]. After 21 days of incubation, irrigation with PBS three times, and stabilization with 4% paraformaldehyde for 10 min, the cells were incubated to use primary antibodies to RUNX-2 and fibronectin (1:100, Abcam, Cambridge, England) overnight at 4 °C. After incubation with primary antibodies, cells were incubated with secondary Alexa Fluor 594 goat anti-rabbit and mouse (Invitrogen, CA, USA) for 1 h at room temperature. The cells were mounted with DAPI mount for 10 min, and the cells were washed with PBS. We confirmed the differentiation of osteoblast cells with colocalization by expression of DAPI, RUNX-2, and fibronectin under high-powered magnification via a confocal microscope (Olympus, Tokyo, Japan).

**Fig. 1:**
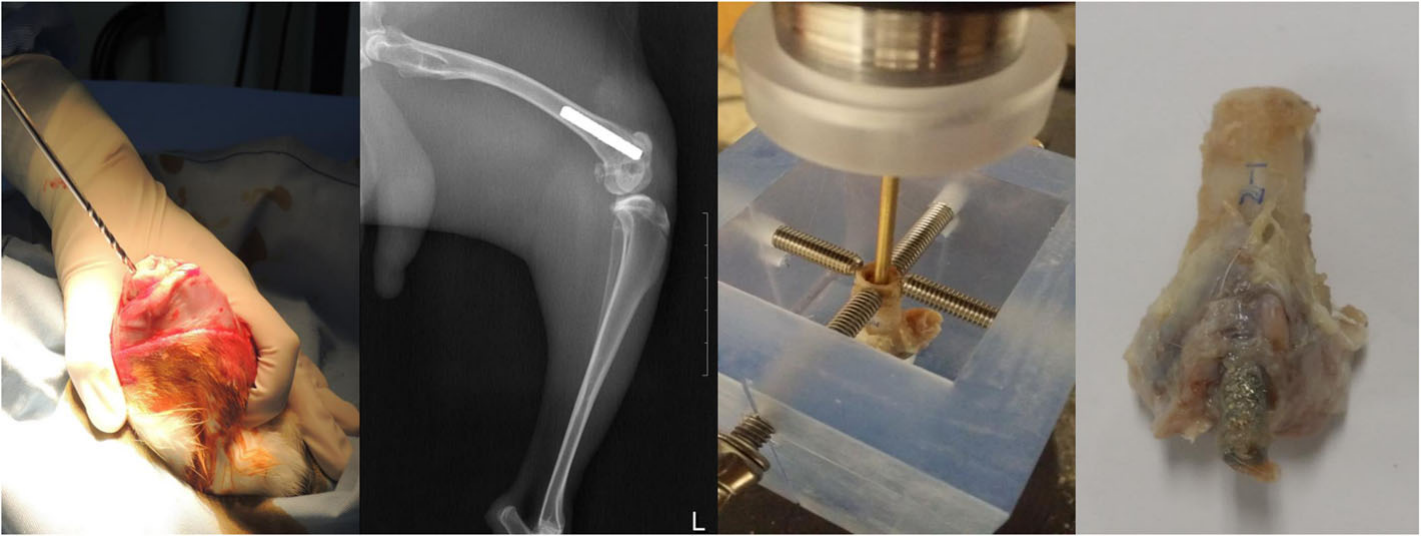
DMF- and TPS-coated rods are inserted separately in the rabbit distal femur and then harvested 3 months after, for in vivo biomechanical analysis. Rods were connected to a jig for push-out test until the femur breaks or the rod comes out

For in vivo study, 20 full-grown rabbits (> 3.2 kg) were assigned as experimental subjects. All experimental procedures were approved by the Institutional Animal Care and Use Committee of the institution. DMF- and TPS-coated rods were inserted surgically into intramedullary canals of each distal femur separately. Specimens were harvested 3 months after the surgery and push-out test, and histomorphometric analysis was conducted. Each harvested distal femur was sliced at the two ends of the rods, and foreign substances were removed. To test the shear strength of the bone-implant interface of the products, a jig of the universal testing machine (Daekyung tech DTU-900MH30kN, Korea) was positioned vertically along the long axis of the rod and then a push-out test was performed at a push rate of 1 mm/min (Fig. 1). The push strength was recorded until the rod became dissociated with the femur or breakage of the femur occurred [3].

The harvested bone tissue was dehydrated with alcohol in stages and soaked in Technovit 7200 resin (Heraeus Kulzer, Germany). The soaked tissue was embedded in paraffin for curing via a light system (Exakt, Germany). The block was sliced into 200-μm-thick sections with a hard tissue slicer (Struers, Germany). These sections were then stained with hematoxylin and eosin (H&E; Sigma-Aldrich). Microscopy images were obtained by ×12.5, ×40, and ×100 (BX51, Olympus, Japan). The specimens from each implant were analyzed by determining the percentage of direct contact between the mineralized bone and the CoCr alloy surface from intersection counting, using an integrative eyepiece with parallel sampling lines at a magnification of ×100 [21].

For statistical analysis, we compared the cell proliferation assays on the two surfaces, mean interfacial shear strength and bone-to-implant contact percentage of the two different surfaces using a Wilcoxon signed-rank test. Statistical analysis was performed using SPSS® 18.0 software (SPSS, Inc., Chicago, IL).

## Result

### In vitro

**Fig. 2:**
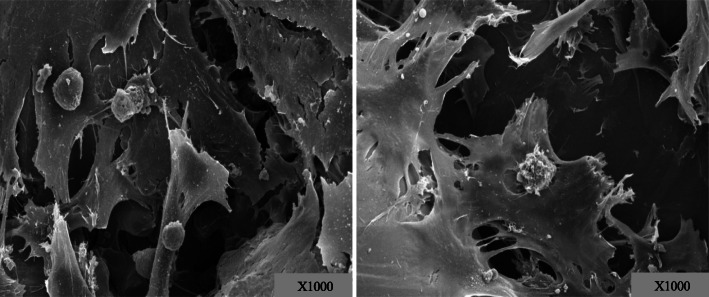
Cell morphology. SEM images of the surfaces of the TPS (left) and DMF (right) coat (×1000). Both surfaces showed similar characteristics. Osteoblast adhesion to surface with lamellipodia and filopodia was visible which means that the surfaces provided environment osteointegration

For cell morphology observation, both TPS- and DMF-coated surfaces were covered with osteoblast which means cell adhesion appeared extensive on both groups (Fig. 2). Lamellipodia and filopodia, a cytoplasmic projection extending into porous structure, formed on both surfaces.

**Fig. 3:**
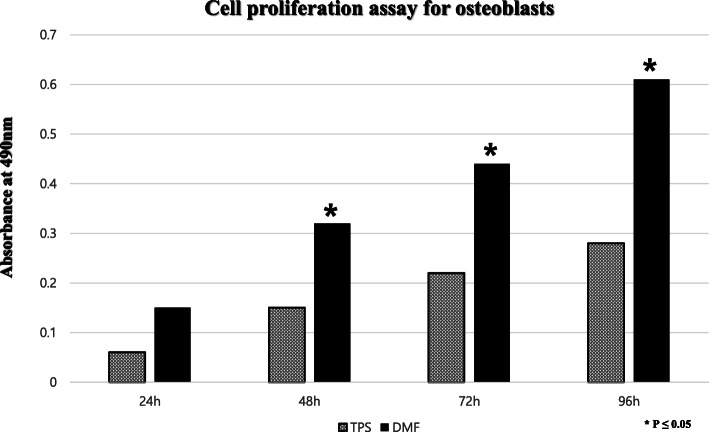
Cell proliferation. ELISA of the surfaces of TPS and DMF coating. As to the proliferation rate of the osteoblasts, the DMF group showed a better result, superior to the plasma-sprayed group on 24, 48, 72, and 96 h of incubation (*p* < 0.001)

**Fig. 4:**
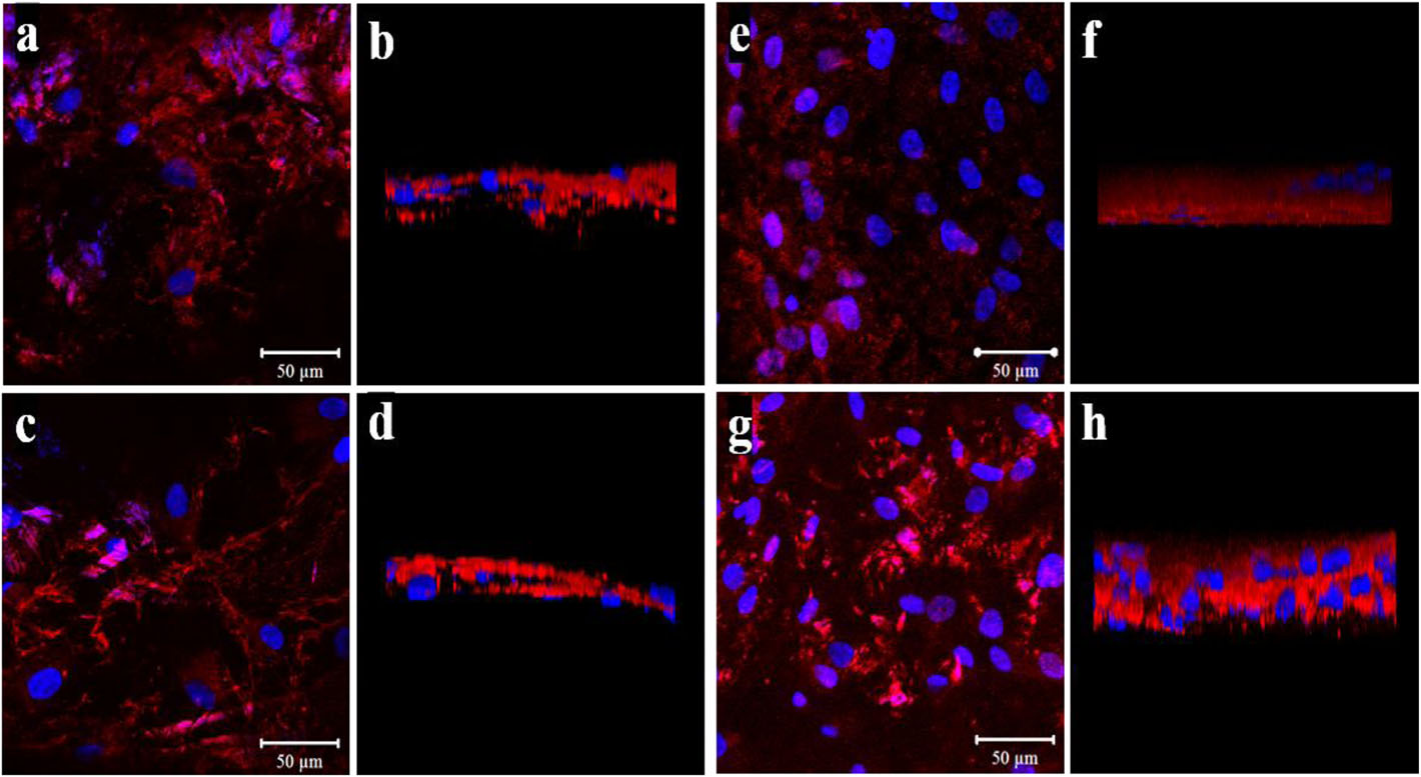
Immunofluorescent staining of Runx-2 and fibronectin expression in osteoblasts. Stained with reds are Runx-2 and fibronectin. TPS-coated surface stained with fibronectin is showed in **a** and **b** while fibronectin staining of DMF surface is showed in **c** and **d**. Runx-2 expression of TPS surfaces is showed in **e** and **f** while that of DMF surface are showed in **g** and **h**. Blue stains are of DAPI, which were used as counterstain. Overall expression within the set area is shown in **a**, **c**, **e**, and **g**. The thickness of the stained layer is shown in **b**, **d**, **f**, and **h**

Cell proliferation on both surfaces was evaluated with ELISA. The number of viable cells was measured at 490 nm (Fig. 3). As to the proliferation of the osteoblasts, the DMF group showed a better result; optical density of 0.15, 0.32, 0.44, and 0.61 at 24, 48, 72, and 96 h while the TPS groups showed 0.06, 0.15, 0.22, and 0.28. Differences were statistically significantly higher in the DMF group at 48, 72, and 96 h (*p* = 0.035, 0.005, and 0.001, respectively). For biocompatibility assay to validate the differentiation and proliferation of osteoblasts, immunofluorescent staining with antibodies to Runx-2 and fibronectin were conducted (Fig. 4). Expression and distribution of fibronectin and Runx-2 genes showed a similar degree of expression on both surfaces.

### In vivo

**Fig. 5:**
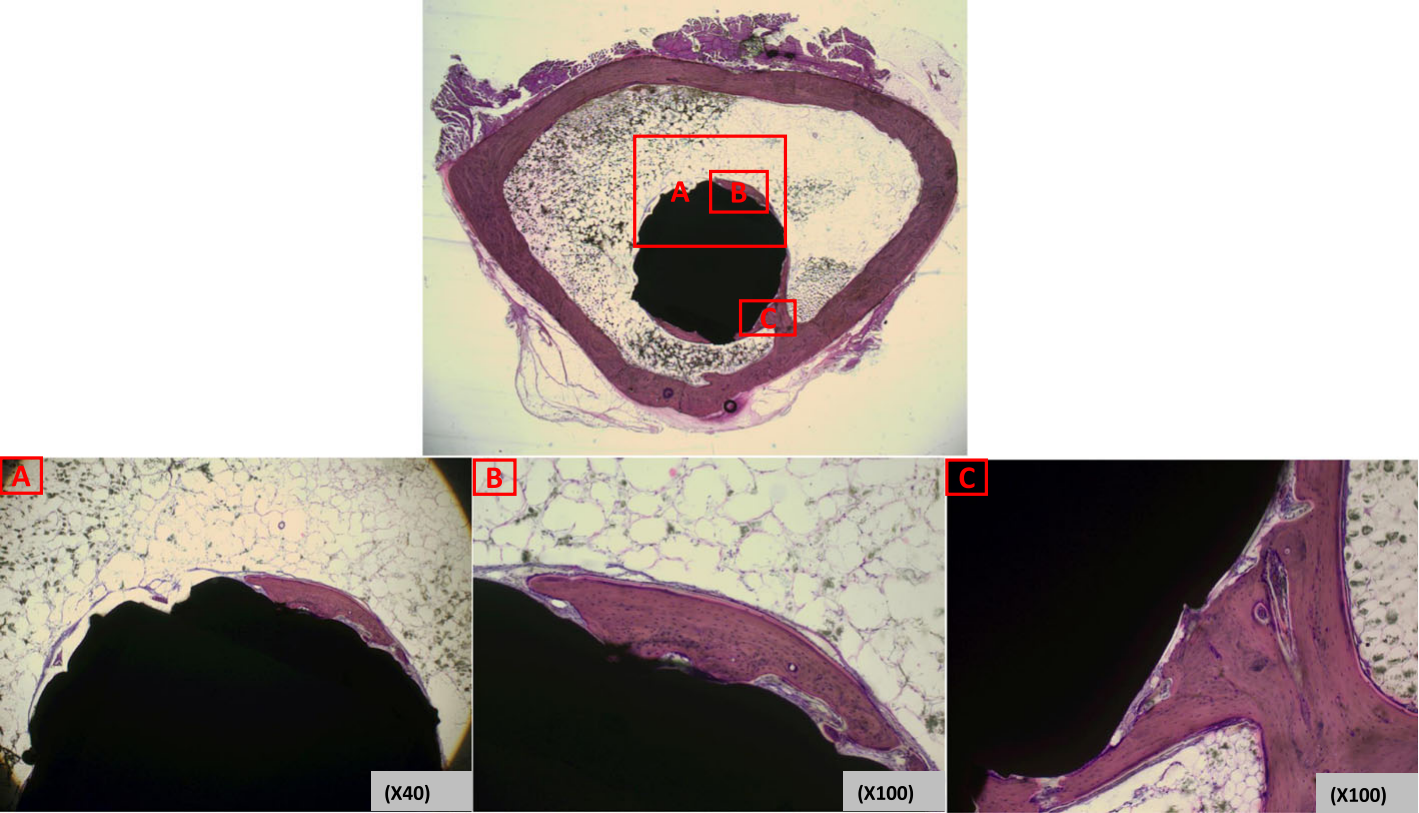
Histomorphometry of bone-implant cross-section. Cross-sections of the bone to implant contact areas **a**, **b**, and **c** are observed. Cellular matrix component stained with hematoxylin is on the contact surface of the implant

The biomechanical push-out test resulted in 2.46 MPa from the TPS rod and 2.53 MPa from the DMF rod (*p* = 0.714). Histomorphometric analysis showed that harvested rods from the rabbit yielded the bone to implant contact ratio of 56.4 ± 6.7% and 57.3 ± 7.2%, from TPS and DMF, respectively (*p* = 0.657) (Fig. 5).

## Discussion

Our study investigated whether DMF could yield a comparable titanium-coated surface to that of TPS. We hypothesized that a novel 3D printing method utilizing additive manufacturing can provide the titanium-coated surface in terms of biocompatibility, osteointegration, and biomechanics both in vitro and in vivo, as competent as a product created by the established method of TPS. As for in vitro, the coated surfaces from both methods created a porous structure similar to that of the cancellous bone which could provide foothold for osteoblast [22]. Adhered osteoblasts displayed cellular projectiles such as lamellipodia and filopodia as we could observe from cell morphology on the scanning electron microscope. As to whether those osteoblasts were proliferating on the surfaces, ELISA was conducted for quantification and found that both surfaces allowed osteoblasts for proliferation. However, absorbance at 490 nm shows that the proliferation rate of the osteoblasts on the DMF surface was higher, compared to the plasma-sprayed group on 24, 48, 72, and 96 h after incubation (*p* < 0.001). Runx-2 and fibronectin expression are specific to osteoblast [20, 23]; thus, an immunofluorescent assay for Runx-2 and fibronectin was conducted for visualization and validation of osteoblast proliferation. Runx-2 and fibronectin were both expressed with a similar degree of the signal intensity in DMF- and TPS-coated surfaces. This shows that biocompatibility and osteointegrative quality were achieved in both surfaces. As for in vivo study, a push-out biomechanics study to test the shear strength of the implant and its histomorphometry was conducted. The push-out test resulted in 2.46 MPa from TPS and 2.53 MPa from DMF (*p* = 0.714). It reveals that there is no statistically significant difference between 2 rods in terms of shear strength. Histomorphometric analysis showed that harvested rods from the rabbit yielded the bone to implant contact ratio of 56.4 ± 6.7% and 57.3 ± 7.2% (*p* = 0.657), which renders this result as statistically not significant. Our results of in vivo and in vitro study show that DMF- and TPS-coated surface were similar in biocompatibility, osteointegration, and mechanical strength. While plasma spraying is already an established method for surface coating, it has some recognized shortcomings such as the requirement for a vacuum environment for processing and difficulty to adjust to various angulation and curvature of the surface of a welded plane. DMF has advantages over TPS that it does not require such manufacturing conditioning and can be more fine-tuned as to powder application, undercooling of welding metal and to curved surfaces. The fact that the whole process can be automated with 3D printing technology is also an advantage that it can be utilized for the personalization of implant design. This study has a few limitations. First, it was not conducted in a clinical setting and thus could not conclude the real applicability of the coating method. Second, our sample size for in vivo study was relatively small and was in the animal study. This renders a need for a further study focused on clinical application in a larger scale. Nonetheless, the result of our study shows that a novel, DMF method is applicable to implant surface coating and that it can be an alternative to the previously existing coating method.

## Conclusion

In conclusion, DMF is a novel method which can be utilized in the creation of Ti-CoCr alloy. The resulting product of the alloy shows a similar quality to that of TPS, both in vivo and in vitro, in terms of biological and mechanical properties. Moreover, DMF applied with 3D printing technology has an advantage over the conventional TPS method in creating alloy surfaces where the curved surface poses technical challenges due to the conformation. We believe this method could be used to create metal surfaces of orthopedic implants with osteointegrative and biocompatible quality.

## Data Availability

All data generated or analyzed during this study are included in the published article.

## Abbreviations

Ti: Titanium
CoCr: Cobalt-chromium
ASTM: American Society for Testing and Materials
CPTi: Commercially pure titanium
DMF: Direct metal fabrication
TPS: Titanium plasma spraying
ELISA: Enzyme-linked immunosorbent assay
